# Why Should Health Researchers Use Policy Theories? Comment on "Modelling the Health Policy Process: One Size Fits All or Horses for Courses?"

**DOI:** 10.34172/ijhpm.2023.7977

**Published:** 2023-07-24

**Authors:** Paul Cairney

**Affiliations:** Division of History, Heritage, and Politics, University of Stirling, Stirling, UK

**Keywords:** Health Policy, Public Policy, Policy-Making, Policy Theories

## Abstract

Powell and Mannion suggest that ‘health policy process’ research should draw more lessons from ‘the wider policy process literature.’ While health research could continue with sector specific models, the wider literature is ‘conceptually stronger.’ In that context, I clarify how and why health researchers should use policy theories. I describe a review of the use of policy theories in public health research to show that many researchers use them to not only understand policy-making but also influence policy and policy-making. Most policy theories are not designed for that purpose, but it is still possible to produce practical lessons. I outline the issues that arise when repurposing theory-informed insights, such as that policy change takes a long time, and the scale of policy-making is potentially overwhelming. I then highlight the valuable role of theories in raising dilemmas in relation to modes of governance and evidence production.

## Introduction

 Powell and Mannion^1^ explore the extent to which ‘health policy process’ research can and should draw more insights from ‘the wider policy process literature.’ Their review of reviews finds that very few health studies provide an awareness of that wider field or a clear indication of how and why they use policy theories. While health research could continue to develop and apply health policy-specific models, they recommend the greater appreciation and application of the wider literature, which tends to be ‘conceptually stronger’ and based on far more empirical studies.

 In that context, I explore the potential obstacles to, and payoffs from, the greater use of policy theories in health policy research. First, I ask: what would health researchers be trying to do, and why? For example, do they simply seek better explanations for the sake of their scholarly understanding? Or, do they want to use that knowledge to improve health policy and policy-making? Key sources in Powell and Mannion’s review suggest that both aims may be conflated with reference to the term ‘policy analysis.’ Second, I describe what the combination of theoretical and normative aims may look like by drawing on a review by Cairney et al of the use of policy theories in public health research (studies of ‘Health in All Policies,’ HiAP).^2^ This review identifies similarly low engagement with policy theories, but also the attempt by some scholars to translate theoretical insights into strategies to influence policy processes. A focus on this search for ‘practical lessons from policy theories’ helps to identify what they can and cannot tell us, and to raise dilemmas regarding the trade-offs between the aims of health researchers.

## If Using Policy Theories, What Would Health Researchers be Trying to Do, and Why?

 To answer this question, it is essential to clarify (1) what using policy theories means, and indeed (2) what policy theories are. First, using policy theories can relate to two distinct aims:

1. To understand policy-making by describing and explaining policy processes.
2. To understand policy-making, then use that knowledge to evaluate or seek to influence policy change.

Powell and Mannion focus primarily on the former, to describe a collection of ‘theories of the policy process’ that inform a programme of empirical studies. However, they also cite the ‘pioneering work of Walt and Gilson’^3^ which focuses primarily on the latter. Walt and Gilson relate this task to damaging reforms in ‘developing countries’: (*a*) economic crisis and ‘shifts towards neo-liberal values’ prompted many countries to reduce public health spending, increase healthcare charges, and subject the health sector to new public management reforms (while relying more on the private sector); and (*b*) ‘negative effects of health reforms on health status, especially on the vulnerable.’ In that context, they criticize a sole focus on the technical content of policy reforms (as if policy could be designed in a vacuum), in favour of a greater understanding of how reforms will fare during implementation (including the power of the actors involved). This understanding is essential to efforts to improve health, since — for example — a vague global commitment to public health principles or strategies (now summed up by the phrase ‘Health in All Policies’) will be futile unless its advocates understand how those commitments will be enacted or undermined by the politics and policy processes of each country.

 Second, in the ‘policy sciences,’^4^ policy theory is not synonymous with ‘policy analysis.’ Instead, there are two distinct but – hopefully – mutually informative types of study:

1. ‘Policy analysis’ describes the (research informed but clearly political) act of defining a problem, generating feasible solutions, using values and goals to compare them, predicting their effects, and making recommendations.
2. ‘Policy process research’ describes the study of policy-making, such as to identify the environment or context in which policy analysis and choice takes place.^5^

Advocates of the ‘new policy sciences’^6^ recommend a return to treating both concerns as symbiotic, to reverse a long-term trend towards the ‘unnecessary split between basic and applied research.’ In other words, for the most part, most of the policy theories listed by Powell and Mannion tend not to be applied to ‘policy analysis.’

 Therefore, disambiguation matters because the use of policy theories in ‘health policy analysis’ can range in meaning, from (*a*) a sole focus on the scientific study of processes, to (*b*) the combination of scientific and applied research. Further, the latter role can include attempts to (*a*) inform policy analysis, (*b*) evaluate the progress or success of a policy strategy or instrument, and/or (*c*) influence policy and policy-making.

## What Practical Lessons Can Policy Theories Provide and not Provide?

 It is straightforward to use elements of policy theories to *describe* or *explain* key concerns in health research. First, for example, we can address classic questions — such as why is there such an absence of ‘evidence-based policy-making’? — to the concept of ‘bounded rationality,’^7^ in which policy-makers do not have the ability to gather and process all policy relevant evidence or to relate it to a coherent set of policy preferences. Rather, they use cognitive shortcuts to make efficient choices.^8^ Health researchers often describe a technocratic solution, to produce more high-quality evidence to reduce policy-maker *uncertainty*, which is incomplete without a political solution based on how policy actors exercise power to reduce *ambiguity* (in other words, the multiple ways to interpret the same problem).^9^ Indeed, the latter concern is a routine feature of HiAP research, in which researchers contrast a damaging ‘neoliberal’ framing of health at the expense of a proper focus on the ‘social determinants of health.’^2^

 Second, a collection of concepts to describe the ‘policy-making environment’ or ‘system’^10^ helps to explain why the adoption of a new policy *strategy* would not lead to the desired policy *outcomes*. They suggest that there is no such thing as a linear and orderly ‘policy cycle’ in which a single powerful central government can simply define a problem, adopt a solution, then guarantee implementation. Rather, there are many policy-makers spread across many levels and types of government (polycentric or multi-centric policy-making), and each venue or ‘centre’ has its own: formal and informal rules (institutions), relationships between policy makers and influencers (networks), dominant ways to understand policy problems and establish the feasibility of solutions (beliefs, ideas, and paradigms), and responses to social and economic conditions or events. Therefore, a decision reached in one centre may be amplified or dampened in others.

 It is far less straightforward to use this knowledge to *respond* to these dynamics. There are some notable attempts to translate theoretical insights into practical lessons in HiAP research,^11,12^ to produce recommendations including:

- Reframe the health and health inequalities problem (as socially determined, not the fault of individuals) and seek audiences in government that are relatively sympathetic to policy change.
- Form alliances, coalitions, or networks of actors who support the social determinants frame and can oppose advocates of neoliberal approaches.
- Support policy entrepreneurs who can exploit windows of opportunity for policy change.

However, in each case, policy theories inform cautionary tales regarding:

- *The time it takes to reframe problems or adopt new solutions*. Classic accounts of multiple streams analysis^13^ and punctuated equilibrium theory^14^ describe such changes as taking place over decades (if at all).
- *The scale of required activity*. A key aim of HiAP advocates is to foster intersectoral action across (and outside of) government. Approaches such as the advocacy coalition framework^15^ describe the spread of policy-making across a huge number of subsystems, each with their own actors, rules, and relationships.

*The limited role of exceptional actors*. While Kingdon^13^ signalled the important role of policy entrepreneurs, they were akin to surfers waiting for the big wave. In other words, their environments provided most of the explanation of policy change opportunities.

 In many ways, policy theories are better suited to more reflective lessons, such as to highlight the dilemmas and trade-offs that emerge from contradictory aims. For example, Cairney and colleagues’ review^2^ identifies two evergreen issues: (1) a *governance dilemma*, when actors seek the benefits of policy-making centralisation (to institutionalise a strategic plan and oblige change) *and* decentralisation (to foster local creativity and collaboration); and, (2) an *evidence dilemma*, when actors seek the benefits of high quality scientific evidence (restricting participation to experts and technocrats) *and* the experiential knowledge of professionals, citizens, and communities (maximising participation and rejecting hierarchies of knowledge).

## Conclusion

 I agree with Powell and Mannion: the study of health policy processes should be informed routinely by insights from ‘the wider policy process literature,’ including the mainstream policy theories that they describe. However, health researchers need to be clear on what they are using these theories for. In other words, to maximise the value of this endeavour, researchers need to know what exactly policy theories are and are not, how and why they seek to use these insights, and if policy theories were designed for this purpose. First, the most direct value of theories comes from their ability to help explain health policy processes. Policy theories provide abstract insights that can be applied empirically in different ways to specific contexts Examples include to identify how and why policy-makers pay attention to and interpret policy relevant evidence in particular ways (such as through a ‘neoliberal’ lens), the extent to which policy-making environments are conducive to the implementation of specific health strategies, and if one strategy (such as for public health) may be undermined by another (such as when healthcare commands far more attention and resources). Second, policy theories can aid the pursuit of policy analysis, such as to encourage the designers of policy instruments to consider how (*a*) they would interact with the existing policy mix, and (*b*) the extent to which their success depends on collaboration across multiple policy-making centres. Third, however, they do not offer simple practical lessons on how to respond, such as to improve the use of some evidence or help to produce more coherent policy or joined-up policy-making. Rather, they may help to provoke more critical reflection on the dilemmas and trade-offs associated with policy-making.

## Ethical issues

 Not applicable.

## Competing interests

 Author declares that he has no competing interests.
